# 

**Published:** 1893-01

**Authors:** 


### CONTENTS—JANUARY, 1893—VOL. VIII., NO. 7.

ORIGINAL COMMUNICATIONS—

Report of Eighteen Cases of Typhoid Fever. Professor H. A. West, M. D., Galveston . . . . . . . . . . . . . . . . . . . . . . . . . . 249
Discussion by Galveston County Medical Society . . . . . . . . . . . 253
Paralysis Agitans. A lecture by Prof. A. J. Smith, M. D., Galveston. 260
Laparotomy for Fibro-Cystic Tumor. R. E. Houghton, M. D., Midland, Texas . . . . . . . . . . . . . . . . . . . . . . . . . 269

THERAPEUTIC NOTES—Dr. David Cerna, Galveston.

Pyoktanin in Cancer, Carbuncle, Glycerine in Hepatic Colic, Iodozon in Phthisis; Diaphtherin Thiol for Burns, Phenolsalyl. Influence of size of dose. Active principles of Cod Liver Oil. . . . . . . . 275

EDITORIAL—

Higher Education . . . . . . . . . . . . . . . . . . . . . . . . . . 281
A Bill to Regulate the Practice of Medicine in Texas . . . . . . . . 281
The Public Health Bill . . . . . . . . . . . . . . . . . . . . . . . 284
Typhus Fever in Mexico . . . . . . . . . . . . . . . . . . . . . . 285

BIOGRAPHICAL—

Dr. David Cerna . . . . . . . . . . . . . . . . . . . . . . . . . . 286

MEDICAL NEWS AND MISCELLANY . . . . . . . . . . . . . . . . . 287
BOOK NOTICES . . . . . . . . . . . . . . . . . . . . . . . . . . . 291
PUBLISHER'S NOTES . . . . . . . . . . . . . . . . . . . . . . . . 294

